# c-MYC and Epithelial Ovarian Cancer

**DOI:** 10.3389/fonc.2021.601512

**Published:** 2021-02-26

**Authors:** Jeyshka M. Reyes-González, Pablo E. Vivas-Mejía

**Affiliations:** ^1^Center for Collaborative Research in Health Disparities, University of Puerto Rico, Medical Sciences Campus, San Juan, Puerto Rico; ^2^Department of Biochemistry, University of Puerto Rico, Medical Sciences Campus, San Juan, Puerto Rico; ^3^Comprehensive Cancer Center, University of Puerto Rico, San Juan, Puerto Rico

**Keywords:** ovarian cancer, MYC, undruggable oncogenes, targeted therapies, biomarkers

## Abstract

Ovarian cancer is the deadliest of gynecological malignancies with approximately 49% of women surviving 5 years after initial diagnosis. The standard of care for ovarian cancer consists of cytoreductive surgery followed by platinum-based combination chemotherapy. Unfortunately, despite initial response, platinum resistance remains a major clinical challenge. Therefore, the identification of effective biomarkers and therapeutic targets is crucial to guide therapy regimen, maximize clinical benefit, and improve patient outcome. Given the pivotal role of c-MYC deregulation in most tumor types, including ovarian cancer, assessment of c-MYC biological and clinical relevance is essential. Here, we briefly describe the frequency of c-MYC deregulation in ovarian cancer and the consequences of its targeting.

## Introduction

Ovarian cancer is the most lethal gynecologic malignancy with an estimated 21,410 new cases and 13,770 deaths expected for 2021 in the United States (1). According to the tissue of origin, ovarian tumors are classified into epithelial and non-epithelial types (2). Tumors that arise from germ and sex cord stromal cells in the ovaries constitute ~10% of ovarian cancers (3). Epithelial-derived ovarian tumors account for ~90% of ovarian cancers and can be subdivided into four major histological subtypes including serous, endometrioid, clear-cell, and mucinous carcinomas (3). Of these types, high-grade serous tumors (HGSOC) are the most commonly diagnosed (3). Despite advances in surgical and therapeutic options for ovarian cancer, resistance to platinum-based chemotherapy remains a major clinical challenge. Several mechanisms of platinum resistance have been proposed, including the altered expression of oncogenes such as c-MYC (4, 5).

c-MYC was discovered four decades ago as the human cellular homolog of the avian myelocytomatosis viral oncogene (v-myc) (6–10). Further studies strongly linked c-MYC to cancer, marking it as a bona fide human oncogene (11, 12). Following the initial discovery of c-MYC, genomic amplification of two additional human paralogs N-MYC and L-MYC were identified in neuroblastoma and small-cell lung cancer, respectively (13–15).

Oncogenic c-MYC arises through multiple molecular mechanisms at the DNA, RNA and protein levels, rendering c-MYC no longer dependent of control signals (16–19). c-MYC deregulation reprograms gene expression and promotes uncontrolled cell proliferation – one of the hallmarks of cancer (16, 20–22). Given its pivotal role as a driver in cancer progression and maintenance, as well as its association with drug resistance, c-MYC has become an ideal target for cancer therapy (19, 22, 23). However, given the lack of enzymatic activity and the absence of surface domains suitable for most pharmacological inhibitors, c-MYC is considered an “undruggable” protein (24, 25). Nevertheless, several strategies have been employed to inhibit c-MYC transcription, disrupt c-MYC/MAX dimerization, or prevent binding of c-MYC/MAX heterodimers to enhancer box (E-box) DNA sequences (19). Antisense oligonucleotides and RNA interference (RNAi) directed against c-MYC, as well as, inhibitors targeting c-MYC upstream and downstream signaling pathways have also been evaluated (19).

## c-MYC Function and Regulation

c-MYC is a basic helix-loop-helix leucine zipper (bHLHZ) transcription factor that regulates the expression of ~15% of all human genes (26). Binding of c-MYC to promoter regions of target genes at E-boxes (including the 5′-CACGTG-3′ consensus sequence and other non-consensus sites) requires dimerization with its protein partner, MAX (27, 28). Upon DNA binding, c-MYC/MAX heterodimer recruits co-factors required for transactivation of gene expression (26, 29). As a transcription factor, c-MYC plays a central role in the control of several essential functions including proliferation, growth, cell-cycle progression, angiogenesis, metabolism, differentiation, apoptosis, cell adhesion and motility, among others (16, 20, 26, 30–32). In addition, c-MYC may repress gene expression through interaction with MIZ-1, SP1/SP3, and NF-YB/NF-YC transcription factors (33).

In normal (non-transformed) cells, c-MYC expression is tightly regulated at multiple levels (34). Transcriptionally, c-MYC is controlled by numerous transcription factors (including CNBP, FBP, and TCF), enhancers, and non-B DNA structures such as G-quadruplexes (35, 36). Post-transcriptional regulation of c-MYC is exerted by RNA-binding proteins (CELF1 and HuR) and non-coding RNAs (35, 37). Post-translationally, c-MYC stability and transcriptional activity are controlled by a variety of different proteins (33). Phosphorylation at Serine 62 (Ser62) by Ras-activated ERKs stabilizes c-MYC and promotes activation (38). Subsequent phosphorylation at Threonine 58 (Thr58) by GSK3β leads to PP2A-mediated dephosphorylation at Ser62 and ubiquitination by Fbw7, resulting in c-MYC proteosomal degradation (38). Oncogenic activation of c-MYC is commonly induced by gene amplification or translocation, transcriptional upregulation, and enhanced protein stabilization (16, 35).

## c-MYC Deregulation in Ovarian Cancer

### c-MYC Gene Amplification

c-MYC is located in chromosome 8q24, which is frequently translocated or amplified in cancer (39). In fact, integrated genomic analyses of ovarian carcinoma revealed that one of the most common focal amplifications resides within the region containing c-MYC (40). Early reports by Yasue et al., using Southern blot hybridization, showed that c-MYC was amplified in human ovarian tumor cell lines (41). Later, Zhou et al. reported c-MYC amplification in 25% of ovarian tumors, mainly papillary serous adenocarcinomas (42). Additional studies found c-MYC amplification in ~20–50% of ovarian carcinomas (43–54). In contrast, Smith et al. found no evidence of c-MYC rearrangement or amplification in tissues from serous adenocarcinomas (55). Nevertheless, Ross et al. identified c-MYC amplification as a potentially targetable genomic alteration in patients with relapsed epithelial ovarian cancer (EOC) (48).

By using fluorescent *in situ* hybridization (FISH) on ovarian tumor tissue arrays, Dimova et al. reported a high frequency for c-MYC copy-number increases (38.5%), including 22.1% amplifications and 16.4% gains (47). In addition, c-MYC copy-number changes were associated with the degree of malignancy and histological type (47). Similarly, by using next-generation sequencing (NGS), Du et al. found that c-MYC had a high frequency of copy-number variations (29%) in tumors from recurrent ovarian cancer patients (56). Surprisingly, by using quantitative PCR (qPCR) analysis, Yamamoto et al. observed significantly higher c-MYC copy-numbers in early-stage EOC, however, low c-MYC copy-numbers were associated with a statistically significant poor prognosis (57).

Darcy et al. found limited predictive or prognostic value of c-MYC gene amplification and polysomy for chromosome 8 in women with suboptimally-resected, advanced-stage EOC (58). In contrast, Wang et al. reported a trend toward poorer survival for ovarian cancer patients with c-MYC amplification (51). In fact, survival was significantly poorer in patients with amplification of both HER-2/neu and c-MYC oncogenes (51). Similarly, a study by Katsaros et al. found that patients with c-MYC amplification and high p185/p21 co-expression had a significantly worse survival than those with normal levels (52). Moreover, Jung et al. reported an association between c-MYC amplification with late stage and high grade in endometrioid EOC (59). However, c-MYC amplification had no impact on clinical outcome in serous and endometrioid tumors (59). Diebold et al. found no correlation between c-MYC amplification and histological tumor type, histological grade, FIGO stage, DNA ploidy, proliferative activity or prognosis (50). Similar results by Baker et al. showed no apparent relationship between c-MYC amplification and tumor grade, response to platinum-based chemotherapy, hormone receptor status, or initial CA-125 levels (46). Taken together, these observations suggest that although c-MYC gene copy-number variation and amplification have been commonly reported in ovarian cancer, a relationship between c-MYC gene aberrations and prognostic or clinicopathological significance has not been clearly established.

### c-MYC mRNA Expression

Early studies by Slamon et al., using Northern blotting, showed that c-MYC transcript levels were higher in human ovarian adenocarcinomas compared to normal tissues (60). Similar reports showed that c-MYC mRNA levels were higher in early-stage ovarian cancer tissues compared with those in normal samples, as evident by qPCR analysis (57, 61). In fact, Kohler et al. found that c-MYC mRNA expression was increased in 47.6% of ovarian carcinomas (62). Similarly, a study by Tashiro et al. revealed that c-MYC transcripts were overexpressed in 37.5% of ovarian tumors (including 63.6% of serous adenocarcinomas) relative to normal ovarian tissues (63). Moreover, significantly higher c-MYC expression was observed in stage III compared with stage I and stage IV tumors (63). On the other hand, a study by Bauknecht et al. showed high c-MYC mRNA expression in 28% of ovarian carcinomas (64). An association between c-MYC gene amplification and high mRNA expression levels was also observed (59, 64).

Tanner et al. found no significant association between c-MYC mRNA expression in EOC and clinical parameters including metastatic spread, survival time, FIGO stage, or histological grade and type (65). Similarly, Yamamoto et al. found no significant difference between c-MYC mRNA expression levels and survival rate for early-stage EOC (57). Jung et al. also reported no relationship between high c-MYC mRNA expression and patient outcome in serous and endometrioid tumors (59). On the other hand, a study by Iba et al. comprising EOC specimens from patients who underwent the standard of care revealed that responders had higher c-MYC mRNA levels than nonresponders, and a better 5-year survival rate (66). In contrast, analysis of HGSOC data from The Cancer Genome Atlas (TCGA) revealed significantly worse disease-free (DFS) and overall (OS) survival in patients with high c-MYC mRNA levels (67). Overall, the clinical significance of c-MYC mRNA expression in ovarian cancer has been inconsistent.

### c-MYC Protein Expression

Expression of the c-MYC protein had been previously detected in ovarian carcinoma tumor and stromal cells by immunohistochemical methods (IHC) (62). Using the same approach, Skírnisdóttir et al. observed positive staining for c-MYC in 76% of cases from early-stage EOC (68). Positivity status was associated with tumor grade (68). Similarly, Chen et al. found that c-MYC protein was overexpressed in 65.9% of cases from EOC compared to normal ovary; however, no significant difference was observed between histological subtypes (69). Plisiecka-Hałasa also observed a high incidence of c-MYC overexpression in endometrioid and clear-cell carcinomas (70). By using flow cytometry, van Dam et al. found that c-MYC protein was overexpressed in 35% of epithelial ovarian carcinomas (71). A similar study by Watson et al. showed that serous papillary ovarian carcinomas expressed significantly higher nuclear c-MYC protein levels compared with normal ovary (72).

Reports by Sasano et al. revealed no significant correlation between c-MYC intracellular distribution and nuclear and histological grade or mitotic activity in ovarian carcinomas (73). Nevertheless, studies in ovarian mucinous tumors showed that positive c-MYC protein expression and distribution correlated with tumor size and tumor classification, respectively (74, 75). However, retrospective analysis of clinical data suggested that a standard histological criteria is a more accurate indicator of tumor behavior than assessment of the pattern of c-MYC expression based on immunostaining alone (75).

Paradoxically, Plisiecka-Hałasa et al. found that c-MYC overexpression was associated with better tumor differentiation, higher p27, and lower Ki-67 expression in ovarian carcinomas treated with platinum-based regimens (70). On the other hand, Ning et al. found that increased nuclear c-MYC expression in early-stage ovarian cancer correlated with clinical stage and shorter overall survival (61). However, a study by Curling et al. showed no significant association between c-MYC protein and prognosis in ovarian carcinomas (76). Similarly, Jung et al. found no relationship between high c-MYC protein expression levels and patient outcome in endometrioid tumors (59). Yamamoto et al. also reported no significant difference in survival rate for c-MYC protein expression in early-stage EOC (57). Nevertheless, a positive association between phosphorylated c-MYC (Ser62) and expression of proliferation markers such as Ki-67 was observed (57). In addition, high phosphorylated c-MYC was associated with relatively poor prognosis (57). Similar to amplification and mRNA expression, the association between c-MYC protein levels and clinical parameters in ovarian cancer is not clear. Assessment of the clinical relevance of phosphorylated c-MYC in ovarian cancer warrants further investigation.

## Targeting c-MYC in Ovarian Cancer

### Antisense Oligonucleotides

Early reports showed that targeting c-MYC *in vitro* with triplex-forming (TFOs) and liposomal phosphorothioate oligonucleotides (PTOs) inhibits ovarian cancer cell growth (77, 78). In fact, evidence indicates that PTOs against c-MYC inhibit the proliferative effect of TGFα in ovarian cancer cells (79). Also, resistance to TGFβ – an antiproliferative growth factor – coincides with the loss of c-MYC repression in ovarian carcinoma cells (80). On the other hand, Janicek et al. showed that PTOs against c-MYC in ovarian cancer cells leads to both antiproliferative and stimulatory activity (81).

### Small Interfering RNAs (siRNAs)

SiRNA-mediated c-MYC knockdown in MYC-amplified ovarian cancer cells inhibits proliferation and induces replicative senescence by increasing the Cdk inhibitor p27 and decreasing CDK2 activity (82). High c-MYC, low p27, and high phosphorylated Rb protein signature correlates with poor patient survival in ovarian cancer (83). Induction of p27 by miR-124 decreases phosphorylated Rb and c-MYC protein levels leading to cell cycle arrest *in vitro* and reduced tumor growth *in vivo* (83). Moreover, targeting c-MYC with siRNAs in platinum-resistant ovarian cancer significantly inhibits cell growth and viability, induces cell-cycle arrest and activates apoptosis *in vitro*, and reduces tumor growth *in vivo* (67).

### Small-Molecule Inhibitors

Blocking c-MYC/MAX heterodimerization with the small-molecule inhibitor 10058-F4 significantly inhibits ovarian cancer cell proliferation in part by inducing apoptosis and cell cycle arrest (84). Similarly, 10058-F4 treatment in primary cultures of epithelial ovarian carcinoma induces caspase-3 activity and inhibits cell proliferation (84). Moreover, c-MYC inhibition with 10058-F4 reduces glutamine uptake in cisplatin-resistant ovarian cancer cells (85).

Elevated expression of c-MYC has been observed in primary HGSOC cells sensitive to BRD4 inhibition by JQ1, a selective small-molecule BET bromodomain inhibitor (86). By targeting BRD4 and c-MYC, JQ1 suppresses ovarian cancer cell proliferation and induces apoptosis (87). In addition, c-MYC amplified primary cell lines and xenografts derived from chemotherapy-resistant ovarian tumors are sensitive to JQ1 (88). In fact, JQ1 increases the sensitivity of platinum-resistant ovarian cancer cells to cisplatin (87).

Dual targeting of FAK—an integrin-linked non-receptor tyrosine kinase—and c-MYC by VS-6063 and JQ1 inhibitors leads to cell cycle arrest and decreased cell survival in ovarian cancer cells *in vitro* (89). In primary tumors of HGSOC, co-upregulation of FAK and c-MYC suggest a co-targeting approach as a therapeutic strategy in ovarian cancer (89). Residual cells from HGSOC patients treated with neoadjuvant carboplatin and paclitaxel chemotherapy exhibit elevated FAK activity (90). Inhibiting FAK sensitizes platinum-resistant ovarian cancer tumors to cisplatin *in vivo* (90).

Simultaneous inhibition of CDK7 and CDK12/13 with THZ1 abrogates c-MYC expression and decreases tumor growth in platinum- and PARP inhibitor-resistant patient-derived xenograft (PDX) models of HGSOC (91). Dual inhibition of PARP (Olaparib) and CDK4/6 (Palbociclib) inhibits the growth of ovarian cancer cells *in vitro* and slows down tumor growth *in vivo* in part by inducing homologous recombination (HR) deficiency in a MYC-dependent manner (92). Concomitant upregulation of glutaminase (GLS) and c-MYC has been observed in platinum-resistant ovarian cancer cells (93). Inhibition of GLS—a downstream target of c-MYC—by CB-839 sensitizes ovarian cancer cells to PARP inhibition and prolong survival in tumor-bearing mice (93).

### MicroRNAs (miRNAs)

Small non-coding RNAs such as miRNAs have been implicated as regulators and mediators of c-MYC function (37). Therefore, miRNAs may serve as potential therapeutic targets against MYC-driven cancers (37). Lower expression of miR-145 has been observed in EOC cell lines and tumor tissues, and its upregulation inhibits cell proliferation and promotes apoptosis by directly repressing c-MYC (94). In addition, miR-145 inhibits glutamine metabolism in ovarian cancer through c-MYC/GLS1 pathways (95). Furthermore, high miR-145 expression was significantly associated with increased overall survival in patients with ovarian cancer (95). Similarly, EOC tissues and cells exhibit lower levels of miR-494 (96). Overexpression of miR-494 inhibits *in vitro* growth and migration by directly targeting c-MYC (96). Recently, Majem et al. also found that miR-654-5p is downregulated in ovarian serous carcinomas and restoration suppresses ovarian cancer development by impacting on the oncogenic function of MYC, AKT and Wnt pathways through directly targeting CDCP1 and PLAGL2 (97).

Cisplatin-mediated downregulation of miR-145 has been shown to contribute to PD-L1 upregulation in ovarian cancer (98). Increasing miR-145 levels negatively regulates PD-L1 by repressing c-MYC expression in cisplatin-resistant ovarian cancer cells (98). These observations suggest that miR-145 may serve as an adjuvant therapeutic target in ovarian cancer (98). Sun et al. also demonstrated that c-MYC regulates cisplatin resistance in ovarian cancer by suppressing miR-137 and promoting expression of EZH2, which in turn activates cellular survival pathways (99). On the other hand, inhibition of c-MYC-miR-137 axis sensitizes resistant cells to cisplatin (99). Active c-MYC-miR-137-EZH2 was also confirmed in tumor samples from recurrent patients with ovarian cancer (99). Similarly, overexpression of let-7g increases sensitivity to cisplatin treatment in EOC, and inhibits cell growth by c-MYC and Cyclin-D2 downregulation (100). In addition, siRNA-mediated silencing of the histone deacetylase HDAC1 suppresses cell proliferation, increases apoptosis, and sensitizes ovarian cancer cells to cisplatin treatment by inducing c-MYC downregulation and miR-34a upregulation (101).

### Long Non-coding RNAs (lncRNAs)

Evidence indicates that lncRNAs are able to control the expression and function of c-MYC (102). In addition, c-MYC transcriptionally regulates lncRNA expression through feedback loops (102). For example, c-MYC directly stimulates transcription of DANCR, an oncogenic lncRNA upregulated in ovarian cancer (103). Silencing DANCR increases p21 expression, decreases cell proliferation, and reduces ovarian tumor burden in an orthotopic xenograft model (103). Another oncogenic lncRNA, MALAT-1, which is upregulated in EOC tissues and cell lines, promotes c-MYC mediated epithelial-mesenchymal transition through sponging miR-22 (104). Silencing MALAT-1 inhibits cell proliferation, migration, and invasion (104). On the other hand, MAGI2-AS3, which is lowly expressed in ovarian cancer tissues and cell lines, acts as a tumor inhibitor by negatively regulating miR-525-5p and enhancing MXD1 expression (105). MXD1 competitively interacts with MAX, repressing c-MYC transcriptional activity (105). These findings suggest that targeting MYC-related lncRNAs may represent a potential alternative therapeutic strategy against ovarian cancer.

## Concluding Remarks

As a transcription factor, c-MYC plays a key role in the regulation of multiple cellular processes. In non-transformed cells, c-MYC expression is tightly controlled. However, aberrant c-MYC expression has been reported in most human tumors. Thus, it is not surprising that c-MYC has been considered as a potential therapeutic target against many cancer types, including ovarian cancer. In fact, several approaches have been proposed to inhibit c-MYC either directly or indirectly, some of which have entered clinical trials. Reports on the prognostic value of c-MYC in ovarian cancer have been inconsistent, which may be explained in part by the complexity of the disease, patient background, and choice of methodology. Further investigation into the potential role of c-MYC as a prognostic marker in ovarian cancer is required in the context of histological subtypes, disease subgroups, genetic racial/ethnic differences, and reliable detection methods.

## Author Contributions

JR-G and PV-M contributed to draft preparation, review, and editing. All authors contributed to the article and approved the submitted version.

## Conflict of Interest

The authors declare that the research was conducted in the absence of any commercial or financial relationships that could be construed as a potential conflict of interest.
